# The dopamine dilemma: Why scientific conferences must evolve

**DOI:** 10.1016/j.jacig.2026.100788

**Published:** 2026-09-22

**Authors:** Don Bukstein, Marcus S. Shaker, Ray S. Davis

**Affiliations:** a Problem Based Learning Institute, LLC, Chesterfield, Mo; b Allergy, Asthma, and Sinus Center, Milwaukee, Wis; c Departments of Medicine and Pediatrics, Geisel School of Medicine at Dartmouth, Hanover, NH; d Dartmouth-Hitchcock Medical Center, Section of Allergy and Immunology, Lebanon, NH

**Keywords:** Problem-based learning, postgraduate medical education, team-based learning, interprofessional education

## Abstract

Human learning is facilitated by human connection. This rostrum discusses the need to evolve traditional didactic continuing medical education to more interactive learning models to advance postgraduate medical education.

Imagine you are settling in for the morning plenary of a national medical meeting. You see hundreds of your colleagues, leaders in the field, esteemed specialists—who have traveled across the country, registered months in advance and paid for hotel rooms. A guru is on stage presenting mind-boggling breakthroughs regarding data in genetic reprogramming of cells.

Within 10 minutes of the monotone lecture by the guru, you scan the room and realize you are not in a temple of knowledge; you are in a quiet warehouse of digital distraction.^1^, ^2^, ^3^, ^4^, ^5^ More than half the audience is scrolling through Facebook, Instagram, or other apps, doing bargain retail shopping, or checking patient messages on Epic, whereas many others are catching up on their rapid eye movement sleep in the midst of arguably the most exciting research developments in your field.^2^, ^6^, ^7^, ^8^ Although some are drafting tweets or taking notes, many of those on their mobile applications are chasing a different kind of stimulation—the instant, personalized thrill of “pocket dopamine” (ie, their cell phone).^9^, ^10^ This phenomenon is not our collective fault; it is a profound, inevitable symptom of a 21st-century addiction.^11^ The guru is getting the new cutting-edge information OUT “to the masses” with a typical barrage of data dumping from one study to the next in rapid succession whose effect resembles a white noise bedside sleep machine to those attendees who are catching up on their sleep due to either jet lag or awakening so early for this seminar.^12^ The bottom line is that a rare few are absorbing all of the esteemed guru’s information!^12^ Notably, the continuing medical education (CME) structure may not be “wrong” and may still have value, but for many, passive learning through lecture can be difficult. Although many speakers are very engaging—as evidenced by the success of TED (Technology, Entertainment, and Design) talks—interactive learning is often absent in didactic formats. As adult learning theory suggests, oftentimes passive learning is relegated to rote memorization in our gray matter and may not affect how clinicians’/learners’ behavior will change when applying the “new knowledge,” which is one of the major goals of CME.^2^, ^13^, ^14^ Interactive group learning approaches can help to activate engagement. A summary of group learning methods is shown in Table I.^15^

**Table I:** Group learning methods

Group learning method	Features	Example
Problem-based learning (PBL)	PBL is used in undergraduate and graduate professional education. It consists of a “case open” portion in which the group works through a case in steps. Problems are identified, discussed, and debated. The “case close” portion of the activity may occur at some later time to allow self-directed learning, may take place immediately following the PBL activity through a discussion from a facilitator-turned-teacher, or be presented in a blended fashion through a didactic lecture.	PBL has been used as an established instructional method in medical schools across the country. It has also been used across multiple specialties in postgraduate venues, including the Eastern Allergy Conference for nearly 2 decades.
Team-based learning (TBL)	In TBL, groups of learners approach a complex problem using teamwork to solve questions and develop plans in real time. A TBL approach is characterized by an individual-readiness assurance test and a group-readiness assurance test.	TBL provides experience in negotiating teams and managing a variety of competing perspectives. It has primarily been used at the undergraduate level, but some forms of postgraduate PBL incorporate a TBL-like component approach but without the individual-readiness assurance test and group-readiness assurance test.
Interprofessional education	An interprofessional experiences approach involves learners with backgrounds from complementary disciplines, including nursing, physician assistant, and medical students.	It is important to appreciate that clinical team members have broad backgrounds and training. Developing and nourishing the ability to work together as interprofessional teams is paramount to providing safe and effective health care.

Reproduced with permission from Shaker et al.^15^

After a quarter-century of maintaining a stagnant, archaic lecture format, it is time to admit that the traditional conference didactic, delivered exclusively without an interactive format, is becoming obsolete when used as the sole meeting content.^7^, ^8^, ^16^ Although didactic lecture can be a foundational component, true learning happens through connection.^12^, ^17^

To be effective, lectures should follow an engaging interactive case discussion with the attendees/participants, such as done in a problem-based learning (PBL) case discussions.^14^, ^15^, ^18^, ^19^, ^20^ Then, the lecturer could more effectively “fill the information gap” of the participants following a “real time needs assessment” by a skilled PBL facilitator if they are properly trained to do so.^1^, ^2^ This has been referred to as “blended PBL” or lectures “in context” of a PBL case discussed just minutes before. It gives the participants immediacy of the “need to know” updated guidelines in context to the case/patient whom they could likely see the following week in their clinic.^1^, ^2^, ^7^, ^15^

## Why? The cognitive clash: Old brains, new stimuli

The “digital revolution” has not merely introduced new tools; it has rewired our cognitive processing.^9^, ^10^ Constant exposure to screens and short-form content has fostered a reduced patience for effortful, long-form attention.^21^ Research into “TikTok brain” suggests that heavy exposure to short, rapid-fire content leads to weaker focus and increased difficulty staying on tasks requiring sustained concentration.^22^ A traditional 45-minute monologue, regardless of how “revolutionary” the science is, cannot compete with the instantaneous dopamine reward of social media.^1^, ^2^, ^9^, ^17^, ^19^, ^21^, ^23^ “Cute babies talking videos” are simply easier to process than complex regulatory gene manipulation when the brain is exhausted by travel, which will prevent sustained attention to even a “guru” lecturer!^20^, ^24^

The brain of the “new millennium” seems a bit different from the brain of the 20th century.^25^, ^26^ Or, is the constant stimulus just different?^5^, ^9^, ^10^, ^22^

## The myth of the highly motivated audience

We often assume that a large, expensive in-person conference inherently fosters higher engagement.^27^ We believe that because people “made the effort” to get there, they will naturally be attentive.^28^ However, that assumption ignores the reality of modern work culture.^29^, ^30^ The modern attendee is often in the middle of a high-pressure job.^31^ They are often checking messages or dealing with Epic or work-related urgent tasks.^32^, ^33^ Furthermore, digital distractions may be a major factor in online and in-person professional meetings, with multitasking becoming the norm rather than the exception.^10^, ^29^, ^34^, ^35^, ^36^, ^37^, ^38^

The value derived from a live lecture must exceed what can be obtained via a Zoom webinar or a recorded lecture.^39^ If the content is merely a passive “talk” that is recorded and available later, the rationale for in-person attendance falls apart.^15^, ^40^, ^41^ Why sit in a dimly lit, cold ballroom to watch a screen when you can watch it at 2× speed at home, without the distractions of the person next to you browsing social media?^27^, ^34^, ^40^ To make in-person meetings matter again, they must offer, as some recent trends suggest, “immersive, authentic experiences that connect them to the destination’s culture, people, and stories.”^42^ Group learning approaches such as PBL can also help to facilitate shared decision-making skills, which are crucial to the implementation of conditional recommendations.^15^, ^43^, ^44^

## Reimagine and modernize: The path forward using PBL

For over 50 years since Harvard and McMaster medical schools first introduced PBL to undergraduate medical students, PBL has spread to virtually every US medical school and many abroad as well.^45^ It has been shown to effectively change physician behavior toward accepting and following new guidelines and new therapies, which is what the goal of most CME courses is supposed to be.^2^, ^15^, ^46^, ^47^, ^48^ Lectures alone are less effective at changing physician behavior.^46^, ^47^, ^48^ Yet, we continue to pack the masses into large plenary rooms and kid ourselves into believing that this is effective postgraduate medical education. But it is NOT!

So, how do we fix this? A roadmap to change begins with an understanding that connection and interaction are the heartbeat of human learning and change.^2^, ^15^, ^19^, ^40^, ^49^ Practical steps forward include:

• including sessions with a more engaging, interactive, format focus such as PBL for CME conferences.^2^, ^8^, ^18^
• developing case-based, focused learning (with trained facilitators), which resonates with attendees who become “participants” in interactive case discussions.^4^, ^50^
• training more PBL facilitators to create PBL cases based on “needs assessment” CME evaluation forms to help laser-guide learning to those health care professionals who admit their shortcomings and want to fill their information gaps by discussing cases with their peers—often learning pearls from each other.^51^, ^52^
• creating “post-PBL lectures” in context of the case just discussed before the lectures so that the “brief didactic lectures” are not just abstract lectures without a case context discussed just before the lectures.^2^, ^7^, ^13^, ^16^, ^53^
• emphasizing the importance of communication, compassion, and use of shared decision-making with our patients.^54^, ^55^, ^56^ Shared decision-making is critical for implementation of conditional recommendations and its skills are fostered by group learning approaches (Fig 1).

**Fig 1:**
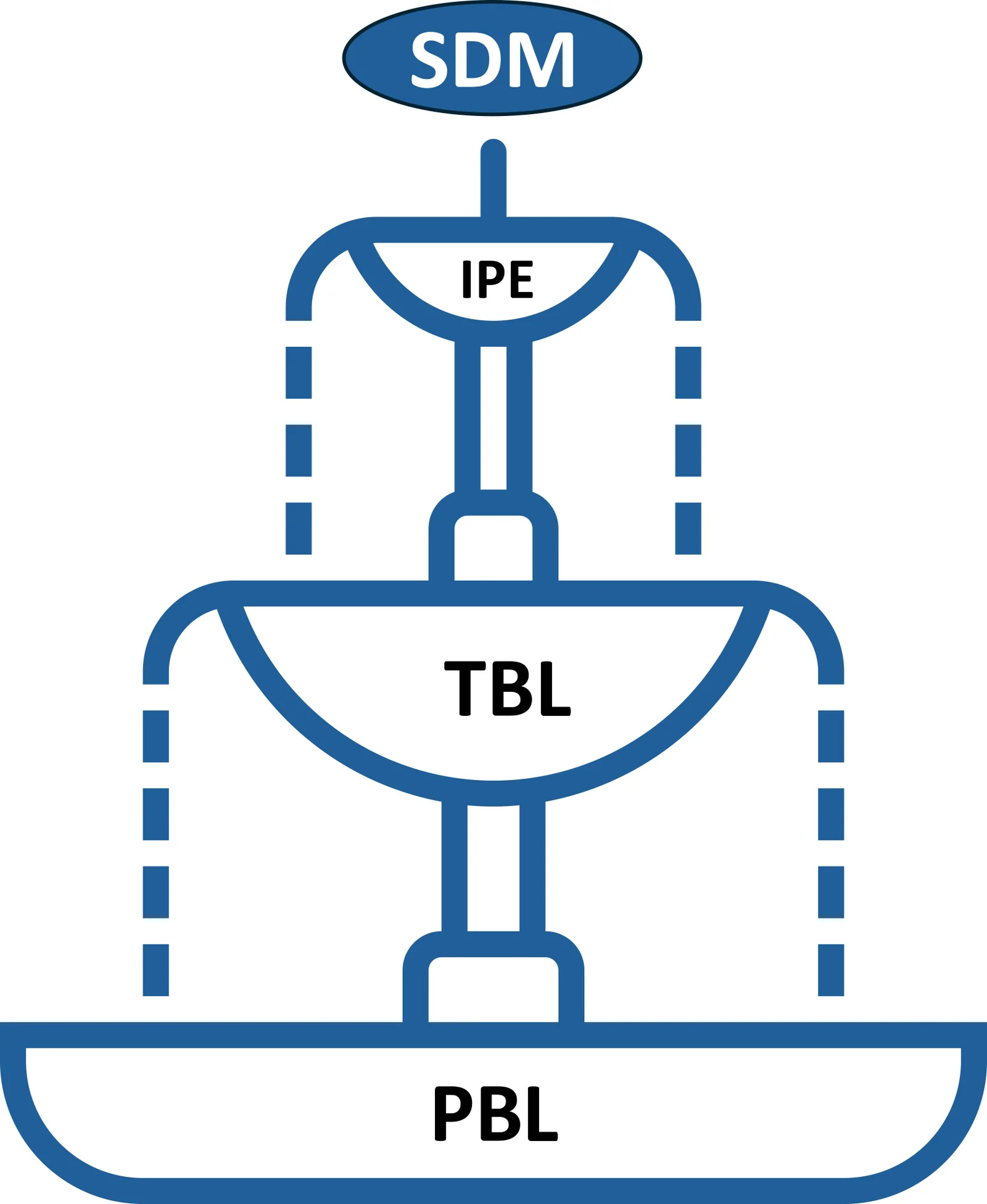
Synthesis of group learning and shared decision-making. Group learning approaches such as the keystone of problem-based learning (PBL) help foster curiosity, connection, and humility—paramount traits of successful clinicians and the bedrock of true adult learning theory. PBL and group interactive learning can foster shared decision-making (SDM) approaches and act as a “fountain of youth” by building stronger relationship skills. In moving beyond simple instruction and helping clinicians learn to cocreate care plans with patients, these methods can result in abilities to deepen patient-clinician trust and help to combat misinformation. IPE, Interprofessional experiences; TBL, team-based learning. Reproduced with permission from Shaker et al.^15^
• leveraging storytelling.^57^ The most engaging post-PBL lecturers/speakers are not just knowledgeable; they are entertainers.^1^, ^19^, ^25^ They use humor and narrative to deliver their science, making the information stick “in context” of a PBL case that has just been discussed.^2^
• encouraging deliberate networking over passive consumption.^58^ Instead of having 500 people listen to one person speak, a plenary could be redesigned to have 500 people discuss, brainstorm, or network about the topic in smaller, facilitated PBL groups.^59^, ^60^
• focusing PBL education on the “Why” and “What Next”; or “What should I do differently with a similar patient on Monday morning to follow the newest practice parameters which I may not have been utilizing as I should have?”^2^, ^15^ Keep knowledge acquisition contemporary and up to date.^61^, ^62^, ^63^
• remembering the power of connection.^18^ “What do the rest of you think?” is a powerful expression of open-mindedness, humility, honesty, and willingness to learn.^43^, ^44^

We must remember that a tradition popularized by the famous Dr William Osler at Johns Hopkins Medical School was that medical students should learn by bedside rounds, listening to patient histories, examining them, and discussing their possible diagnoses and treatments with their peers and an attending.^64^, ^65^, ^66^ This was also a common practice in most medical schools, even in Grand Rounds, until the late 1900s, when it became more popular to just use the new technology of PowerPoint presentations with a didactic lecture.^67^ Importantly, many clinicians learn best through the lens of patient experience.^68^

## Conclusion: The final four of science

The morning plenary of a meeting should be the National Collegiate Athletic Association (NCAA) Final Four for our field—a high-stakes, exciting, must-see event.^69^ Instead, it is too often a lullaby—the treatment for insomnia or an accompaniment to pocket dopamine.^10^, ^70^ We are not failing as professionals because we surf our phone applications; we are adapting to an environment that rewards speed, convenience, and immediate gratification.^11^, ^71^

If we want to keep the in-person meeting relevant, we must recognize that the audience’s brains seem to be wired differently now than they were when PowerPoint hit the scene in the late 1900s.^1^, ^42^, ^57^ They require, and deserve, a format that is as dynamic as the science they have come to learn. The future of the conference should now be more interactive, personal, and more succinct. National organizations such as the American Academy of Allergy, Asthma, and Immunology are recognizing the time for change and are beginning to adopt more interactive learning, based on a true adult learning theory, with a “blended form of PBL.” Such group learning approaches remind us that together we are more effective and sometimes the most impactful learning opportunities present themselves in community.

## Disclosure statement

Disclosure of potential conflict of interest: D. Bukstein has served as a consultant/speaker for Astrazeneca, Regeneron, Sanofi, Kalvista, ARS, and Teva. M. S. Shaker is a member of the Joint Task Force on Practice Parameters, serves on the editorial board of *The Journal of Allergy and Clinical Immunology: In Practice*, is an associate editor of *Annals of Allergy, Asthma, and Immunology*, and serves on the Board of Directors of the American Academy of Allergy, Asthma, and Immunology (views expressed are his own); serves as a strategic medical partner for allergy immunology for Social Theory. R. S. Davis works as a consultant/speaker for Regeneron, Sanofi, Grifols, Kalvista, Astrazeneca, Takeda, Teva, and ARS.

Funding: None declared.
